# Changes in social support and the emotional health of immigrant, refugee, and non-immigrant children across middle childhood: A three year follow up study.

**DOI:** 10.23889/ijpds.v7i3.1923

**Published:** 2022-08-25

**Authors:** Kimberly Thomson, Carly Magee, Monique Gagne Petteni, Anne Gadermann

**Affiliations:** 1 University of British Columbia

## Objectives

Children who are new to Canada have unique circumstances that can be associated with their emotional health. Using linked immigration and child self-report data, we examined associations between changes in children’s social support and emotional health from ages 9 to 12, for immigrant, refugee, and non-immigrant children.

## Approach

A population sample of N = 4664 immigrant, refugee, and non-immigrant children reported on their peer support and school belonging, as well as their emotional health (life satisfaction, self-esteem, sadness), in Grades 4 and 7. Social support and emotional health were measured using the Middle Years Development Instrument (MDI). Migration background including age at arrival, migration class, generation status, and source region, was obtained from the Immigration, Refugees, and Citizenship Canada database, individually linked to MDI records using children’s Personal Education Number and child date of birth. Multi-level modelling assessed associations adjusting for confounders.

## Results

In the linked sample, 19% of children were first- or second-generation economic immigrants (themselves or their parents were born outside of Canada), 8% were family immigrants, and 5% were refugees. Children with refugee backgrounds reported lower life satisfaction and self-esteem and higher sadness in Grade 4 compared to all other groups. Children with immigration backgrounds reported lower life satisfaction and self-esteem and higher sadness compared to non-immigrants. Refugee children had significantly more positive changes in emotional health from Grades 4 to 7 compared to non-immigrants, and significantly more positive changes in social supports. Positive changes in social supports were associated with positive changes in emotional health of similar magnitude for all children, regardless of migration background. Children with refugee backgrounds on average experienced improved emotional health during middle childhood, and changes in peer support partially accounted for these changes.

## Conclusion

Results suggest that children with migration backgrounds enter school with lower emotional health and are likely to benefit from increased social supports. Likewise, incorporating opportunities to build peer relationships and school belonging is likely to benefit all children, regardless of migration background.

